# A pilot study to explore circulating tumour cells in pancreatic cancer as a novel biomarker

**DOI:** 10.1038/bjc.2011.545

**Published:** 2011-12-20

**Authors:** L Khoja, A Backen, R Sloane, L Menasce, D Ryder, M Krebs, R Board, G Clack, A Hughes, F Blackhall, J W Valle, C Dive

**Affiliations:** 1 Clinical and Experimental Pharmacology Group, Paterson Institute for Cancer Research, Wilmslow Road, Manchester M20 4BX, UK; 2 The Christie NHS Foundation Trust, Wilmslow Road, Manchester M20 4BX, UK; 3 School of Cancer and Enabling Sciences, University of Manchester, Manchester Cancer Research Centre and Manchester Academic Health Sciences Centre, Manchester, UK; 4 AstraZeneca Pharmaceuticals, Alderley Park SK10 4TG, UK

**Keywords:** circulating tumour cells, circulating tumour microemboli, pancreatic cancer, ISET, CellSearch, tumour biopsy

## Abstract

**Background:**

Obtaining tissue for pancreatic carcinoma diagnosis and biomarker assessment to aid drug development is challenging. Circulating tumour cells (CTCs) may represent a potential biomarker to address these unmet needs. We compared prospectively the utility of two platforms for CTC enumeration and characterisation in pancreatic cancer patients in a pilot exploratory study.

**Patients and methods:**

Blood samples were obtained prospectively from 54 consenting patients and analysed by CellSearch and isolation by size of epithelial tumour cells (ISET). CellSearch exploits immunomagnetic capture of CTCs-expressing epithelial markers, whereas ISET is a marker independent, blood filtration device. Circulating tumour cell expression of epithelial and mesenchymal markers was assessed to explore any discrepancy in CTC number between the two platforms.

**Results:**

ISET detected CTCs in more patients than CellSearch (93% *vs* 40%) and in higher numbers (median CTCs/7.5 ml, 9 (range 0–240) *vs* 0 (range 0–144)). Heterogeneity observed for epithelial cell adhesion molecule, pan-cytokeratin (CK), E-Cadherin, Vimentin and CK 7 expression in CTCs may account for discrepancy in CTC number between platforms.

**Conclusion:**

ISET detects more CTCs than CellSearch and offers flexible CTC characterisation with potential to investigate CTC biology and develop biomarkers for pancreatic cancer patient management.

Pancreatic adenocarcinoma is the eleventh most common cancer in the United Kingdom (Cancer Research UK, 2011, http://info.cancerresearchuk.org/cancerstats/types/pancreas/incidence/) with a 5-year survival across all disease stages of 2–3%. Medical treatment with single-agent gemcitabine or more recently FOLFIRINOX combination chemotherapy confers a survival benefit with improvement in quality of life measures (Burris *et al*, 1997; Conroy *et al*, 2011). Molecularly targeted therapies however have failed to show an additional advantage to chemotherapy. The addition of erlotinib to gemcitabine resulted in a marginal improvement in overall survival (OS), although no predictive biomarker has been identified to determine which patients benefit most from therapy (Moore *et al*, 2007; da Cunha Santos *et al*, 2010). Moreover, both cetuximab and bevacizumab have failed to demonstrate an improvement over gemcitabine monotherapy (Kindler *et al*, 2010; Philip *et al*, 2010).

The lack of stratified therapies and limitations to new drug target identification in pancreatic cancer are mirrored and underpinned by a paucity of informative biomarkers. Currently, Ca19-9 is the only clinically implemented biomarker used as a diagnostic aid, and as a surrogate marker of response and patient outcome. However, CA19-9 is not applicable to all patients (10% are not within the Lewis blood group) and can be falsely elevated (Ko *et al*, 2005; Ferrone *et al*, 2006; Goonetilleke and Siriwardena, 2007).

Substantial challenges exist in obtaining tissue from pancreatic cancer patients for histological diagnosis, and acquiring pre- and post-treatment tumour biopsies to monitor pharmacodynamic responses during clinical trials of novel treatments are even more difficult. Circulating tumour cell (CTC) analysis may yield prognostic, predictive and pharmacodynamic biomarker information. Moreover, the molecular characterisation of CTCs may provide insight on the process of metastasis *per se* and could facilitate discovery of new drug targets. If CTCs are demonstrated to be useful as a biomarker(s), then ultimately they could represent a less invasive alternative to tissue for diagnosis and treatment management, particularly where biopsy is considered unsafe or unfeasible.

Recent advances in technology have enabled protocol standardisation to enumerate CTCs and allow comparisons between clinical studies (Krebs *et al*, 2010). Circulating tumour cell number, as determined by the CellSearch technology platform (Veridex, LLC, Raritan, NJ, USA), is of prognostic significance in metastatic breast, colorectal and prostate carcinomas, and is FDA approved for prognostication in these clinical contexts (Cristofanilli *et al*, 2004; Cohen *et al*, 2008; de Bono *et al*, 2008). Our laboratory has demonstrated recently that CTC detection by CellSearch also has prognostic significance in lung carcinomas (Hou *et al*, 2009; Krebs *et al*, 2011b).

Detection of a CTC using the CellSearch platform is dependent on CTC expression of epithelial markers, namely epithelial cell adhesion molecule (EpCAM) for tumour cell capture and cytokeratin (CK) for tumour cell confirmation.

As EpCAM-negative CTCs are not detected using the CellSearch platform, and homogeneity of EpCAM expression on pancreatic CTCs was not established, we reasoned that an alternative approach for CTC isolation and enumeration should be investigated. Isolation by size of epithelial tumour cells (ISET) is a filtration-based, marker-independent method of CTC detection based on cell size and morphology. ISET has been used successfully to study CTCs in patients with melanoma, breast, lung and hepatocellular carcinomas (Vona *et al*, 2004; Pinzani *et al*, 2006; De Giorgi *et al*, 2010; Hofman *et al*, 2011; Hou *et al*, 2011).

The primary objective of this prospective, proof-of-principle exploratory study in patients with metastatic and inoperable pancreatic cancer was to examine the feasibility of CTC enumeration across this heterogeneous patient population, comparing the CellSearch and ISET platforms, in order to determine the utility of CTCs as a source of biomarkers in this disease setting. Exploratory end points were to explore whether CTC number holds potential as a prognostic biomarker relating to the clinical end points of progression-free and OS, and to inform the statistical design of future studies. We previously demonstrated substantial heterogeneity in epithelial and mesenchymal marker expression in lung cancer CTCs using ISET (Hou *et al*, 2011). We also sought to explore the hypothesis that pancreatic CTCs also exhibit nonuniform epithelial marker expression, altered during the process of epithelial to mesenchymal transition (EMT) that is associated with loss of cell–cell contacts, tumour cell invasion and metastasis (Polyak and Weinberg, 2009). To extend our assessment of EMT during metastasis, we compared the expression of EMT markers (EpCAM, CKs, E-Cadherin and Vimentin) in pancreatic tumours and in pancreatic CTCs.

## Materials and methods

### Patients

This single-centre prospective study was undertaken at the Christie Hospital, Manchester, UK. Patients with newly diagnosed or progressive metastatic or inoperable adenocarcinoma of the pancreas were eligible, requiring at least a minimum of 6-week treatment-free period. The population chosen was heterogeneous as this study was exploratory as to whether CTCs were detectable in pancreatic cancer *per se*. Histological diagnosis was not mandatory provided a clinico-radiological diagnosis was agreed by a specialist pancreato-biliary multidisciplinary team. Patients with a previous history of carcinomas within 5 years were excluded.

Clinical data were collected for age, gender, site of primary tumour, site and number of metastases, pathological diagnosis, prior treatment received (including surgical intervention and stent insertion), number of lines of treatment, Ca19-9 levels (at baseline and 8–12 weeks after treatment) and clinical outcomes. Overall survival and progression-free survival (PFS) were calculated from time of consent onto the study. The study was approved by the Local Research Ethics Committee. Peripheral venous blood was taken at study enrolment (before the first planned treatment) for the analysis of CTCs using both CellSearch and ISET platforms. The CellSearch platform was acquired before the ISET device and blood samples obtained from the first 19 patients enrolled were analysed using CellSearch only.

### CTC enumeration by CellSearch

Blood samples were collected in CellSave tubes (Veridex), stored at room temperature and processed within 96 h according to manufacturer's instructions. Procedures relating to the use of the CellSearch platform have been previously described in detail (Allard *et al*, 2004). All cell images collected and displayed on the CellSearch gallery were independently reviewed by two analysts (blinded) with any discordant results rereviewed by both analysts.

### CTC enrichment by ISET

The ISET process has been described previously in detail (Vona *et al*, 2000). Briefly, blood (10 ml) was collected into an EDTA tube (Beckton Dickinson, Franklin Lakes, NJ, USA) and within 4 h divided into 1 ml aliquots. Each aliquot was diluted 1 : 10 with red cell lysis buffer (RareCell Diagnostics, Paris, France) and was loaded into an individual well of the ISET filter module (RareCell Diagnostics) consisting of a 10-well plastic reservoir above a polycarbonate membrane perforated with 8 *μ*m cylindrical pores. Blood samples were filtered by attaching the module to the ISET device and applying gentle regulated suction. This process produced 10 discrete ‘6 mm^2^ membrane ‘spots’, on which cells contained within the 1 ml aliquot of whole blood were deposited. Membranes were stored at −20°C.

### Enumeration of CTCs by ISET

Enumeration of CTCs was performed after ISET filtration and negative selection to exclude CD45 positively stained leukocytes by IHC alongside confirmation of characteristic tumour cell morphology. Individual membrane spots were allowed to equilibrate to room temperature before incubating in pH6 citrate antigen retrieval buffer (S1699, Dako, Glostrup, Denmark) in a 99 °C water bath for 40 min. Membranes were washed briefly in tris-buffered saline (TBS) before placing in 0.2% Triton for 10 min. Membranes were washed in TBS and incubated in a 3% solution of hydrogen peroxide in methanol for 30 min. Membranes were washed again in distilled water before the primary CD45 antibody (1 : 30 dilution clone T29/33, Dako) in S0809 antibody diluent (Dako) was added and incubated overnight at 4 °C. Envision Liquid DAB + Substrate Chromagen System (Dako) were used according to manufacturer's instructions to visualise CD45 staining. Counterstaining with 1 × Gill's haematoxylin was performed for 3 min. The membrane spots were mounted on glass slides using Fairmount aqueous mounting medium (Dako) and coverslips were applied. The Bioview duet microscope system (Olympus BX52 microscope (Olympus) and image analysis software, Bioview, Rehovot, Israel) was used to scan membranes at × 40 magnification. Manual image review and scoring of tumour cells was performed by one analyst (blinded).

CD45-negative cells with a high nuclear to cytoplasmic ratio, irregular shaped, hyperchromatic nuclei and diameter >10 *μ*m were designated as CTCs. Four of the 10 membrane spots obtained were used for CTC enumeration. Mean CTC count was extrapolated to 7.5 ml for direct comparison with the standardised CellSearch enumeration procedure. The mean ISET CTC count from four spots was demonstrated to provide a robust CTC count (Krebs *et al*, 2011a; see Supplementary Figure 2).

### Molecular characterisation of CTCs

Molecular characterisation of pancreatic cancer CTCs was performed for the expression of five epithelial or mesenchymal markers. The number of markers analysed was dependent upon the number of membrane spots per patient sample where CTCs were detected. The following IHC analyses were performed using the procotol described above for CD45 and the following antibodies: EpCAM (1 : 100 #MS-144, Labvision, Kalamazoo, MI, USA), anti-CK C-11 antibody to CKs 4,5,6,8,10,13,18 (1 : 100 #MS-149, Labvision), Vimentin (1 : 100, clone v9, Dako) CK 7, as a pancreatic-specific CK marker (1 : 50, clone ov-TL 12/30, Dako) and E-Cadherin (1 : 1000, clone 36/E-Cadherin, BD Biosciences, Franklin Lakes, NJ, USA). EpCAM expression was assessed in 30 patients, Pan CK (C-11) in 19 patients, CK 7 in 25 patients, E-Cadherin in 30 patients and Vimentin in 31 patients.

### IHC of tumour biopsies

Paraffin-embedded tumour blocks were obtained wherever possible to allow correlation of tumour differentiation status with epithelial and mesenchymal marker expression, and to explore which platform might be more suitable for pancreatic CTC detection, that is, whether CellSearch should be able to detect a majority of CTCs that are EpCAM, CK double positive or whether the marker-independent ISET platform would be more appropriate if epithelial markers were likely to be expressed in a minority sub-population of CTCs only. Sections (4 *μ*m) were stained with haematoxcylin and eosin, EpCAM and CK, dewaxed in xylene, and rehydrated using serial washes in 100–70% alcohol. The same IHC protocol and antibodies used for ISET CTC analysis was adopted to determine tumour EpCAM and CK expression in tumour samples. Tumour differentiation and scoring for staining intensity was assessed independently in a blinded fashion, by an experienced histopathologist (LM).

### Statistical analysis

Statistical analysis was performed using SPSS (IBM, Hampshire, UK) for Windows where *P*-values of ⩽0.05 were considered significant. Graphpad prism was used to produce Kaplan–Meier survival curves.

Variables were positively skewed and were log transformed before analysis to stabilise the sample variance and nonparametric tests were used to satisfy the assumptions of variance between the sample groups. The association of CTCs with individual clinical characteristics, including presence of a stent, number of metastatic sites, site of primary, and degree of primary tumour differentiation and performance status were compared by Fisher's exact test or *χ*^2^ test. Univariate survival analysis was performed using the Kaplan–Meier method with data categorised into (median) middle quartiles that were compared using the log-rank (Mantel–Cox) test (serial CTC thresholds were tested as cut off levels to stratify patients), with the median CTC count chosen as the cut off level for all analysis. Survival was calculated from time of study enrolment to progression (PFS) or death (OS). The Mann–Whitney *U* test was used to determine whether CellSearch and ISET were significantly different, that is, they detected different numbers of CTCs.

## Results

### Recruitment and clinical characteristics of patients

Fifty-four patients aged 35–85 years (29 males and 25 females) were enrolled between July 2008 and October 2009. Ten patients had relapsed or progressive disease, 4 patients relapsed following surgery (one of whom had received adjuvant chemotherapy) and 6 patients had progressive metastatic disease having had previous palliative chemotherapy. The remaining patients were newly diagnosed. Fifty-three patients were evaluable by CellSearch, and of these, 31 patients had paired blood samples for ISET assessment and EMT marker expression. Direct comparison of CTC number between the ISET and CellSearch platforms was possible for 27 patients. Patient clinical characteristics are shown in Table 1.

### CTC enumeration using CellSearch and ISET

**Figure 1:**
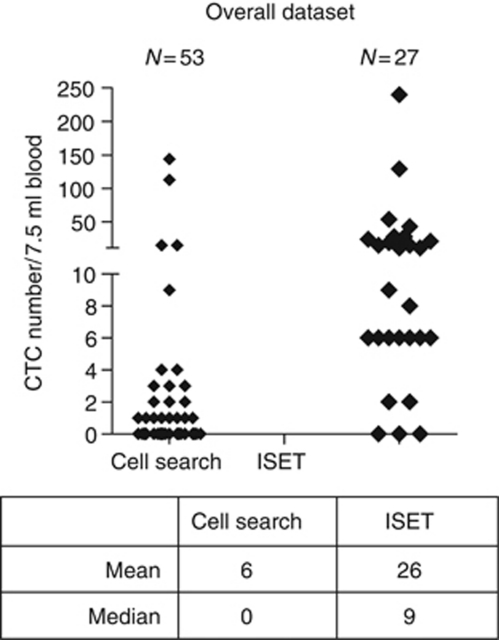
CTC detection by CellSearch and ISET. Comparison of CTC numbers in pancreatic cancer patients using the CellSearch and ISET methods. The number of cells per 7.5 ml blood is shown for all recruited patients, 53 patients had CellSearch analysis and 27 had ISET analysis.

Of the 53 patient blood samples assessed using the CellSearch platform, 21 patients had detectable CTCs defined as ⩾1 CTC (Figure 1). The majority of patients had between 1 and 2 CTCs and five patients had >5 CTCs. Isolation by size of epithelial tumour cells detected higher CTC numbers compared with CellSearch and over a greater dynamic range. The direct comparison of CTC/7.5 ml detected by CellSearch *vs* ISET for the 27 patients with matched samples revealed that the mean CTC/7.5 ml was 2 *vs* 26, the median CTC/7.5 ml was 0 *vs* 9 and that the range was 0–15 *vs* 0–240, respectively. Direct comparison of CTC numbers per patient is shown in Table 2. A nonparametric Mann–Whitney *U* test showed a *P*-value 0.000002 indicating that the CTC numbers detected by CellSearch and by ISET were significantly different to each other.

### CTC number and clinical outcomes

**Figure 2:**
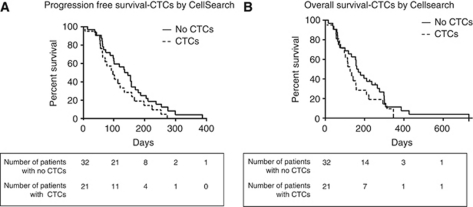
PFS and OS for patients without and with CTCs. (**A**) Progression-free survival of patients without CTCs *vs* those with CTCs using the CellSearch platform. Median survival (days) 140 *vs* 94 hazard ratio 0.63. *P*=0.13 (log rank). (**B**) Overall survival of patients without CTCs *vs* those with CTCs using the CellSearch platform. Median survival (days) 164 *vs* 127 hazard ratio 0.7. *P*=0.26 (log rank).

Compared with those without detectable CTCs, pancreatic cancer patients with a CTC ⩾1/7.5 ml blood, measured by CellSearch, had poorer performance status (50% were performance status 2), higher burdens of disease (54% had more than one site of disease) and had primaries situated in the head or tail of the pancreas (38% and 55%, respectively). However, perhaps due to small sample size none of these variables reached statistical significance (Table 1), and a larger powered study would be needed to demonstrate this formally. The PFS and OS for patients without *vs* those with CTCs was 140 *vs* 94 days (*P*=0.13 log rank, hazard ratio 0.63 (95% confidence interval: 0.34–1.14)) and 164 *vs* 127 days (*P*=0.26 log-rank, hazard ratio 0.7 (95% confidence interval 0.38–1.29)) (Figures 2A and B), respectively. These differences show a nonsignificant trend towards decreased PFS and OS for patients with detectable CTCs (irrespective of what CTC threshold was used) On the basis of these results, 199 patients would be required to determine the prognostic significance of CTC number by CellSearch analysis in a future study.

Circulating tumour cell/7.5 ml measured by ISET (using serial CTC thresholds) did not correlate with either OS or PFS in this small sample set (data not shown). Only nine patients were recruited onto the study with locally advanced disease, but even when these patients were excluded clinical end points of PFS and OS remained non-statistically significant with regard to CTC/7.5 ml.

Univariate Cox regression results for PFS and OS were obtained for CellSearch CTC/7.5 ml, ISET CTC/7.5 ml, baseline and mid-treatment CA19-9 levels, and percentage change in CA19-9 (between baseline and after 8–12 weeks of treatment). Circulating tumour cell/7.5 ml determined using either CellSearch or ISET platforms was not significant for PFS or OS: CellSearch PFS *P*=0.2, OS *P*=0.19; ISET PFS *P*=0.85, OS *P*=0.36. The percentage change in CA19-9 levels was statistically significant for both PFS and OS (*P*=0.0003 and *P*=0.01, respectively) confirming previous studies (Ko *et al*, 2005).

### Expression of epithelial markers EpCAM and CKs in pancreatic tumours

**Figure 3:**
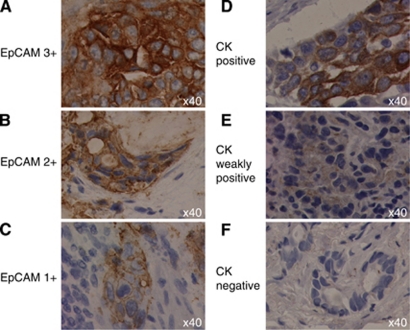
Examples of EpCAM and Pan CK C-11 tumour staining. Examples of staining intensity of (**A**) an EpCAM 3+ tumour, (**B**) an EpCAM 2+ tumour, (**C**) an EpCAM 1+ tumour. (**D**) A CK-positive tumour, (**E**) a CK weakly positive tumour, (**F**) a CK-negative tumour.

We hypothesised that patients whose tumours expresed EpCAM and CK would be more likely to have CellSearch detectable CTCs. Epithelial cell adhesion molecule expression was assessed by IHC in the tumour specimens available from 21 patients within the cohort. The expression of Pan CK (C-11) was also evaluated where sufficient biospy allowed in 13/21 cases. Tumours, reported by a certified pathologist (LM), were graded as either well, moderately or poorly differentiated. All tumours expressed EpCAM; EpCAM expression was graded as 1+ (*n*=6), 2+ (*n*=7) or 3+ (*n*=8) (Figure 3, panels A (3+), B (2+) and C (1+). Cytokeratin expression was graded as positive, weakly positive or negative (Figures 3D–F). There was intratumour heterogeneity in CK expression but only 4/13 tumours were CK positive. There was no correlation between the degree of tumour differentiation, level of expression of EpCAM or CK in tumour and CTC number/7.5 ml by CellSearch. When both the CellSearch capture antigen (EpCAM) and the detection antigen (CK) are expressed in tumour, the CellSearch detected CTC number can be zero. A summary of marker expression is given per patient in Table 3. Positive and negative controls for IHC markers are shown in Supplementary Figure 1.

### EpCAM and CK expressions in CTCs detected by ISET

**Figure 4:**
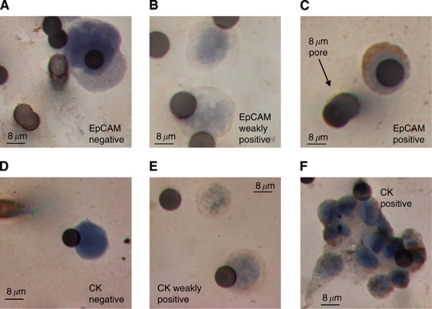
Examples of differential expression of EpCAM and Pan CK C-11 staining of CTCs. (**A**) Epithelial cell adhesion molecule-negative staining in patient 60, (**B**) EpCAM weakly positive staining in patient 54, (**C**) EpCAM-positive staining in patient 51. (**D**) C-11-negative staining in patient 51, (**E**) C-11 weakly positive staining in patient 42, (**F**) C-11-positive staining in patient 50.

The low number of CTCs detected by CellSearch could arise due to low or no CTC expression of EpCAM and/or CK. Of the 30 patients’ CTCs analysed for EpCAM expression, 9 had EpCAM-negative CTCs, 3 had CTCs that were all weakly positive for EpCAM and in the remaining 18 patients EpCAM expression was heterogenous, that is, only a proportion of CTCs were EpCAM postive (Figures 4A–C). Of the 19 patients whose CTCs were analysed for CK, 1 patient had strong expression of CKs in their CTCs, 3 patients had homogeneously CK-negative CTCs, 2 patients had CTCs all of which had weak expression of CKs; (Figures 4D–F) and for the remaining 13 patients their CTCs showed intra-patient heterogeneity in CK expression. However, there was no obvious correlation between the observed CellSearch CTC/7.5 ml and the ISET-detected CTC expression profiles of EpCAM and CKs, which was perhaps not surprising considering that intra-patient heterogeneity in these markers was the predominant finding.

### Circulating tumour microemboli in pancreatic cancer patients

**Figure 5:**
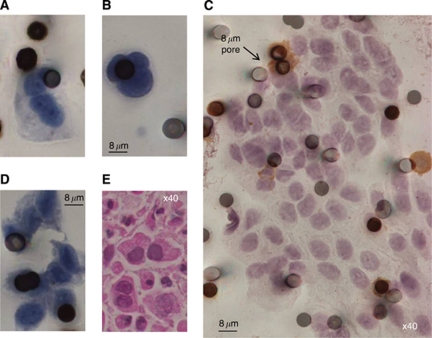
CTCs and CTMs captured on ISET filters. (**A**–**C**) Three different patient samples, filtered through ISET membranes (pores are dark circles) and stained with CD45 antibody so that leukocytes can be excluded; these are shown in brown. Circulating tumour cells were counterstained with haematoxylin. (**C**) A large CTM. The membrane pores are all 8 *μ*m in diameter, which gives scale to these images. (**D**) A CTM from patient 39 (pancreatic head primary and liver, lung and abodminal wall metastases) demonstrates similar morphology to (**E**). (**E**) A haematoxylin and eosin (H&E)-stained tumour block from the same patient photographed at the same magnification.

**Figure 6:**
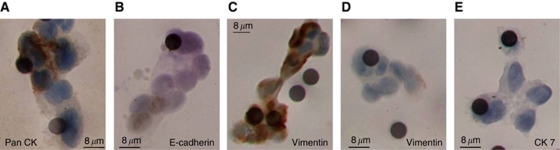
Differential expression of epithelial and mesenchymal markers. CTMs from patient 50 were stained with (**A**) Pan CK (C-11), (**B**) E-Cadherin, (**C**) and (**D**) Vimentin, and (**E**) CK 7. The membrane pores are all 8 *μ*m in diameter, which gives scale to these images.

Tumour cells can circulate in the bloodstream as single cells or in groups termed microemboli. Our previous studies of patients with SCLC showed that circulating tumour microemboli (CTM) were detectable using the ISET but not the CellSearch platform (Hou *et al*, 2009). Similarly, we found that no CTM were observed in pancreatic cancer patients using the CellSearch platform The ISET device detected CTM in three patients, patients 39, 50 and 58, (Figures 5 and 6) where single CTCs were also detected. Pancreatic tumour specimens were available for 2 of 3 patients presenting with CTM: patient 50 had a poorly differentiated EpCAM (3+)-positive carcinoma, with CK-positive cells within CTM by ISET (Figure 6A). Patient 58 had no EpCAM-positive cells within CTM, some CK-positive CTCs by ISET and three CTCs measured by CellSearch. Similar cell morphology was observed when comparing these CTCs and CTM with matched tumour samples (Figures 5D and E). Epithelial to mesenchymal transition is thought to be a dynamic process relevant to CTC extravasation and metastases formation. Cells within CTM were heterogeneous in EMT marker expression (Figures 6A–D). Both CK-positive and -negative tumour cells coexisted within a CTM (Figure 6A), and this within CTM heterogeneity was also observed for both E-Cadherin and Vimentin (Figures 6B–D). Cytokeratin 7 was not expressed in any of the CTCs examined from 10 pancreatic cancer patients (Figure 6E).

## Discussion

Biomarkers are required to develop better therapies for pancreatic cancer patients where access to tumour biopsies is challenging. We undertook a pilot exploratory study comparing the enumeration of CTCs using two technology platforms (CellSearch and ISET) to assess the feasibility of CTCs as a useful source for biomarker research in this disease.

CellSearch detected low CTC numbers in pancreatic cancer patients in comparison with other epithelial carcinomas such as breast, prostate, colorectal and small cell lung cancer (Cristofanilli *et al*, 2004; Cohen *et al*, 2008; de Bono *et al*, 2008; Hou *et al*, 2009) Two previous studies of pancreatic cancer CTCs using CellSearch reported low CTC numbers in agreement with our data. Kurihara *et al* (2008) reported on 26 patients, 11 of whom had CTCs (range 1–105, mean CTC/7.5 ml 17, median, 5) with a mean and median CTC number for all 26 patients of 7 and 0, respectively. Negin *et al* (2010) reported on 48 patients with median CTC/7.5 ml <1, range 0–20. In the Kurihara study, CTC number was significant regarding OS (with *vs* without CTCs 110.5 *vs* 375.8 days, respectively, *P*<0.001) (Kurihara *et al*, 2008); but in the Negin study CTC number did not correlate significantly with OS (with *vs* without CTCs 191 *vs* 269 days, respectively, *P*=0.5) (Negin *et al*, 2010). In our study, ISET detected CTCs in more patients than CellSearch (93% *vs* 40%) and in higher numbers (median CTCs/7.5 ml, 9 (range 0–240) *vs* 0 (range 0–144)), respectively.

The relatively low CTC number reported for gastrointestinal cancers may result from CTC sequestration by the liver as blood passes through the portal circulation into the systemic circulation (Jiao *et al*, 2009; Wind *et al*, 2009). Decreased vascularity is associated with tumour aggressiveness in pancreatic cancer and this may reflect the low CTC number reported (Komar *et al*, 2009; Neesse *et al*, 2011). Primaries in the body and tail of the pancreas are more prone to haematogenous spread than primaries of the pancreatic head (Mao *et al*, 1995), and this is consistent with the data presented here, though further study is warranted to be definitive.

There was no cross-platform correlation in CTC number consistent with their detection of different subsets of CTCs suggested by the heterogeneity of EpCAM and CK expression in both pancreatic cancer patients’ CTCs and tumours and consistent with EMT. This is consistent with findings from two previous studies directly comparing the ISET and CellSearch platforms for CTC detection in resectable non-small cell lung cancer (Hofman *et al*, 2010) and metastatic carcinomas (breast, prostate and lung cancer) (Farace *et al*, 2011). Isolation by size of epithelial tumour cells detected more CTCs and identified Vimentin-positive CTCs in patients who did not have detectable CTCs by CellSearch (Hofman *et al*, 2010). Discrepancies between the platforms appeared to differ between different carcinomas (Farace *et al*, 2011). Epithelial cell adhesion molecule is expressed on normal epithelial cells and overexpressed in a variety of epithelial carcinomas, including pancreatic cancers where heterogeneous EpCAM expression is observed (Baeuerle and Gires, 2007; Trzpis *et al*, 2007; Fong *et al*, 2008; Munz *et al*, 2009). Epithelial cell adhesion molecule expression differs between carcinomas with either a good or poor prognostic indication depending on the carcinoma (Baeuerle and Gires, 2007). The relevance of EpCAM expression to CTC behaviour and metastatic potential is unknown. Epithelial cell adhesion molecule is also cleaved upon activation (Maetzel *et al*, 2009) and this may have implications for CTC capture by the CellSearch kit EpCAM antibody.

Speculative interpretation of the trend seen for CellSearch, but not for ISET regarding clinical outcome, is that the EpCAM/CK-positive CTC sub-population detected by CellSearch is necessary for successful metastases formation as shown in a mouse model (Tsuji *et al*, 2008). Larger studies with balanced patient numbers for each platform will now have to be conducted to assess the prognostic significance of CTC number in pancreatic cancer, and this pilot established that 199 patients will be required to power these future studies.

The identification of viable CTCs by CellSearch is defined as a (minimum) 4 × 4-*μ*m nucleated cell staining positively for EpCAM, CK, and DAPI positivity, and negative for CD45 expression. There are events that do not fit these criteria yet cannot be identified as CTCs despite being CD45 negative. Such events (presumed to be nonviable CTCs or CTC fragments) have been identified in prostate cancer and found to have prognostic significance (Coumans *et al*, 2010). We did not identify any such fragments in this cohort (data not shown). The finding of events classified as CTCs by CellSearch in individuals without cancer is rare. Only 1 out of 344 healthy volunteers and patients with non-malignant diseases had ⩾2 CTCs (Allard *et al*, 2004), and subsequent studies confirmed that ⩾1 CTC in (a presumed) non-cancer population was extremely rare (Cristofanilli *et al*, 2004). Previous ISET studies on healthy populations have been negative for CTCs (Vona *et al*, 2000; De Giorgi *et al*, 2010; Hofman *et al*, 2010, 2011). The identification of CTCs on ISET membranes was based on cell morphology (and compared with primary tumour morphology), CD45-negative selection (for enumeration as leukocytes are the main contaminant, see Supplementary Figure 3) and tumour-associated marker IHC expression, which is consistent with previous ISET studies. It may be that some of these cells are contaminating endothelial or other non-malignant cells. Real-time PCR analysis of nucleic acid extracted from ISET membranes for KRAS for example may support their identification as CTCs, but establishing true tumourigenicity of isolated CTCs would require a mouse model. Simultaneous multimarker assessment of presumed CTCs would also be more discriminatory but is technically challenging on ISET membranes. We have recently developed a four-colour immunofluorescence assay for ISET-filtered CTCs that allows simultaneous analysis of CK and Vimentin, with DAPI-stained nuclei and negative selection for CD45 (see Supplementary Figure 4). In future, this assay will be deployed in a larger study of pancreas cancer patients to explore further the heterogeneity of their CTCs.

To our knowledge, this is the first study to begin to evaluate the ISET platform for pancreatic CTC detection, enumeration and characterisation. Epithelial to mesenchymal transition is thought to occur as cells extravasate into the circulation with the reverse process; mesenchymal to epithelial transition (MET) necessary for cells to intravasate and subsequently form secondary tumours These are most likely to be dynamic events and not necessarily ‘all or nothing’ binary processes. Here, we have begun to reveal EMT marker heterogeneity in CTCs from the pancreatic cancer patients, and a more comprehensive study of their biology with simultaneous assessment of multiple markers is now underway. Epithelial to mesenchymal transition may explain the discrepancies in CTC number between CellSearch and ISET. The finding of both individual CTCs and, more rarely, CTM in pancreatic cancer patients raises questions regarding the mechanisms of cell migration and extravasation, evoking the possibility of collective migration where cells maintain their contacts to invade (Friedl and Wolf, 2003). It remains unclear, however, as to whether tumour cells extravasate as CTM or whether CTM form intravascularly, a phenomenon that has been reported in a preclinical model (Al-Mehdi *et al*, 2000). If CTM form intravascularly this might explain how CTM (containing as many as 50 cells) are found in a peripheral blood sample given that they would otherwise be expected to be trapped within pulmonary capillary beds. Cellular phenotype within CTMs may differ and the overall picture gained from this exploratory study was one of considerable heterogeneity in EMT markers in CTCs and within CTM.

The detection of pancreatic CTCs/CTM on ISET membranes and their downstream characterisation highlight potential for exploiting CTCs/CTM as a surrogate marker of the primary tumour, in a disease where it is notoriously difficult to biopsy tumour diagnostically or to assess tumour evolution or early indication of drug responses. The wider dynamic range of CTC number detected by ISET may be appropriate for pharmacodynamic monitoring to aid dose selection in early clinical trials. The ability to characterise CTCs downstream of ISET filtration in a flexible manner may facilitate measurement of predictive biomarkers allowing stratification of pancreatic cancer patients most likely to benefit from novel targeted therapies, for example, KRAS and EGFR mutation analysis.

Although larger studies are now needed to demonstrate whether the number of CTCs is sufficient for in depth and systemic biomarker assessment, and to exploit the potential of pancreatic CTCs/CTM, this study has highlighted several promising avenues for exploration.

## Figures and Tables

**Table 1:** Clinical characteristics of patients with metastatic or inoperable pancreatic cancer by CellSearch and ISET

**Clinical variable**	**CellSearch CTC>0/total cohort**	**P-value^a^**	**ISET CTC>9/total cohort**	**P-value**
*Stent*				
Absent	13/33 (39%)	0.81	7/14 (50%)	0.85
Present	8/20 (40%)		6/13 (46%)	
				
*Number of metastatic sites*				
0 sites	1/9 (11%)	0.06 (FE)	2/4 (50%)	1 (FE)
1 site	6/18 (33%)		4/9 (44%)	
>1 site	14/26 (54%)		7/14 (50%)	
				
*Site of primary*				
Head	9/24 (38%)	0.41	8/16 (50%)	0.32
Body	1/8 (13%)		2/3 (67%)	
Tail	6/11 (55%)		3/5 (60%)	
>1 site	1/2 (50%)			
No primary	4/8 (50%)		0/3 (0%)	
Head and tail	16/36 (44%)	0.20 (FE)	11/21 (52%)	1 (FE)
				
*Degree of differentiation*				
Well	1/4 (25%)	1 (FE)	0/1 (0%)	0.57 (FE)
Moderate	4/11 (36%)		0/3 (0%)	
Poor	3/7 (43%)		2/4 (50%)	
Moderate and poor	7/18 (39%)		2/5 (40%)	
				
*Performance status*				
0	4/11 (36%)	0.66 (FE)	1/4 (25%)	0.35 (FE)
1	9/26 (35%)		9/15 (60%)	
2	5/10 (50%)		2/7 (29%)	

Abbreviations: CTC=circulating tumour cell; ISET=isolation by size of epithelial tumour cells.

a *χ* test or Fisher's exact test (FE).

**Table 2:** CTC number as detected by CellSearch and ISET for 27 patients

**Patient number**	**CTC CellSearch/7.5 ml**	**CTC ISET/7.5 ml**
31	0	28
32	0	2
33	0	15
34	1	54
35	0	15
36	1	24
37	9	21
39	0	28
40	0	0
41	0	9
42	3	43
44	2	11
45	0	19
46	0	11
49	4	0
50	0	129
51	0	2
52	4	6
54	1	6
55	0	8
58	3	240
60	1	0
61	0	6
64	1	6
65	15	6
66	1	6
67	0	6

Abbreviation: ISET=isolation by size of epithelial tumour cells.

**Table 3:** Degree of tumour differentiation, tumour EpCAM and tumour cytokeratin staining in relation to CTC number by CellSearch (site of biopsy/resection if not pancreatic is given in brackets) for 21 patients

**Degree of differentiation**	**EpCam staining**	**Pan-cytokeratin staining**	**CellSearch CTC number**
Well	1+	Weakly positive	2
Moderate	1+	Insufficient material	0
Poor	1+	Weakly positive	0
Poor	1+	Weakly positive	0
Poor (liver)	1+	Insufficient material	0
Moderate	1+	Weakly positive	15
Poor	2+	Insufficient material	3
Moderate (node)	2+	Weakly positive	0
Well	2+	Insufficient material	0
Moderate	2+	Negative	0
Moderate	2+	Weakly positive	4
Poor (liver)	2+	Insufficient material	1
Moderate	2+	Positive	1
Well (omentum)	3+	Negative	0
Moderate	3+	Insufficient material	0
Moderate	3+	Positive	0
Moderate	3+	Negative	0
Moderate	3+	Insufficient material	0
Poor	3+	Insufficient material	0
Poor	3+	Positive	1
Well	3+	Positive	0

Abbreviations: CTC=circulating tumour cell; EpCAM=epithelial cell adhesion molecule.
