# Supplementary material for: A pilot study to explore circulating tumour cells in pancreatic cancer as a novel biomarker
Source: Br J Cancer. 2011 Dec 20;106(3):508–16. doi: 10.1038/bjc.2011.545 (PMC3273340; doi:10.1038/bjc.2011.545)
Supplement: Supplementary Figures 1–4 [file bjc2011545x1.ppt]

## Slide 1
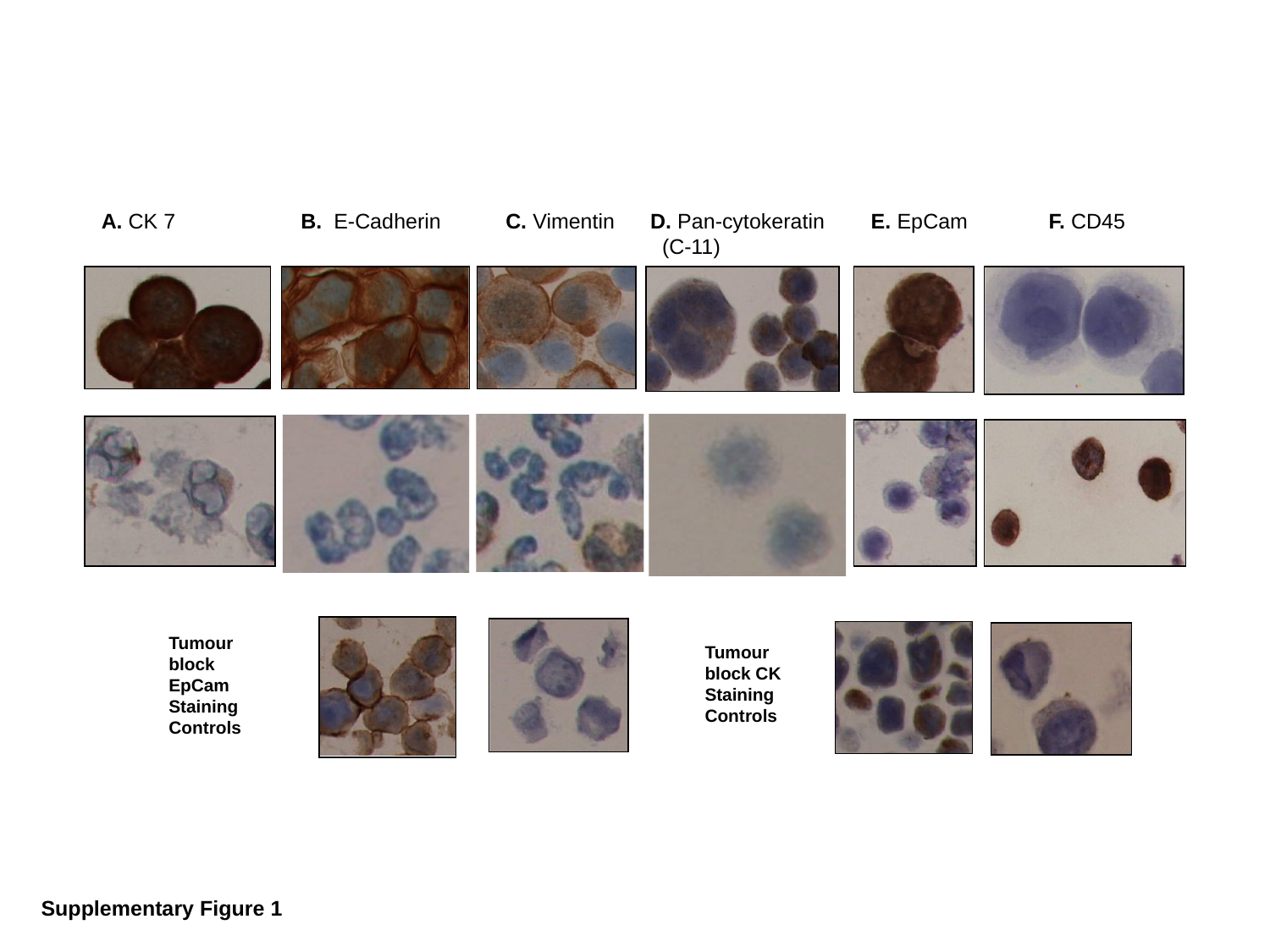

A. CK 7
B. E-Cadherin
C. Vimentin
D. Pan-cytokeratin (C-11)
E. EpCam
F. CD45
Tumour block EpCam Staining Controls
Tumour block CK Staining Controls
Supplementary Figure 1

## Slide 2
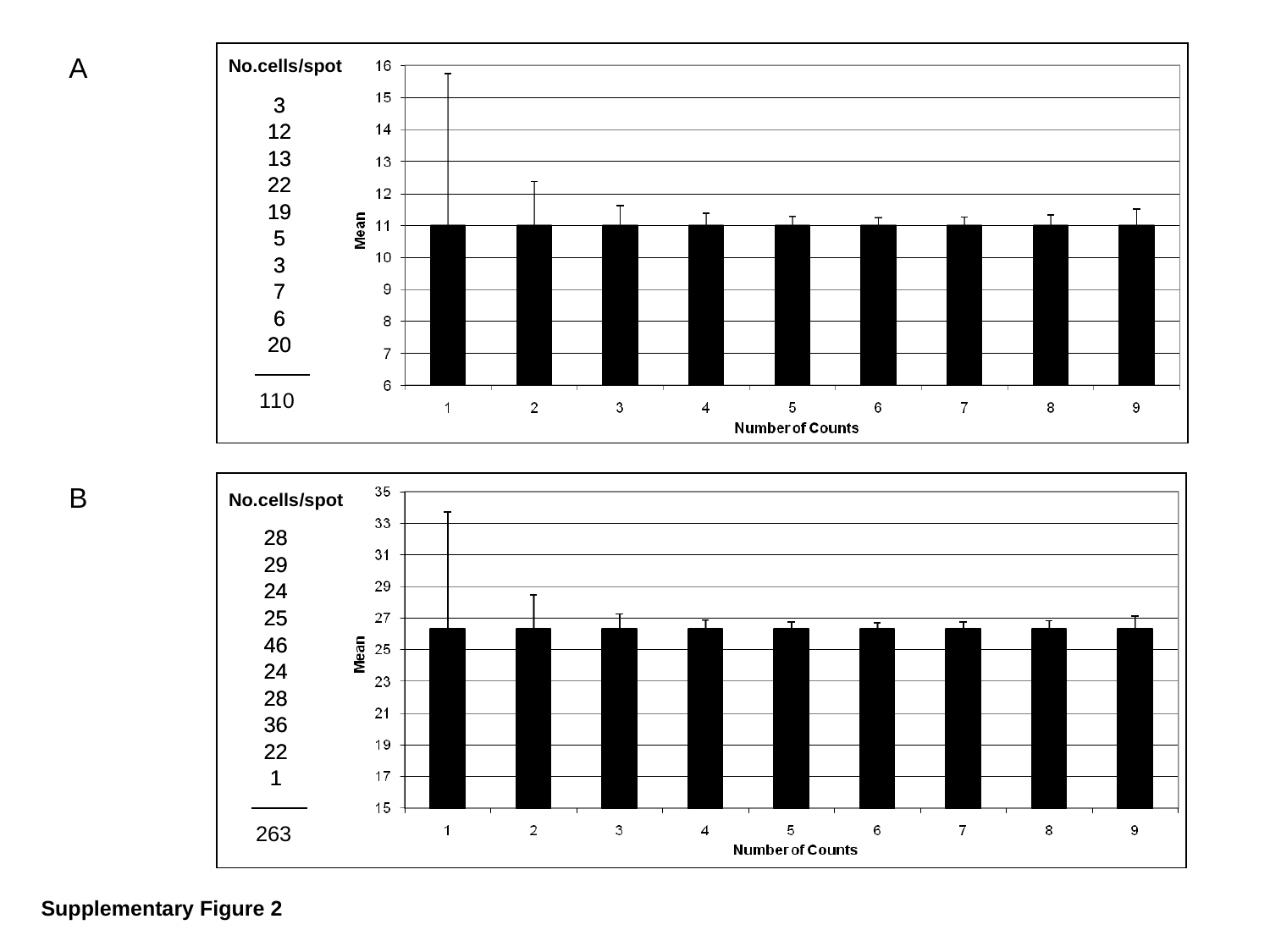

A
B
Supplementary Figure 2

## Slide 3
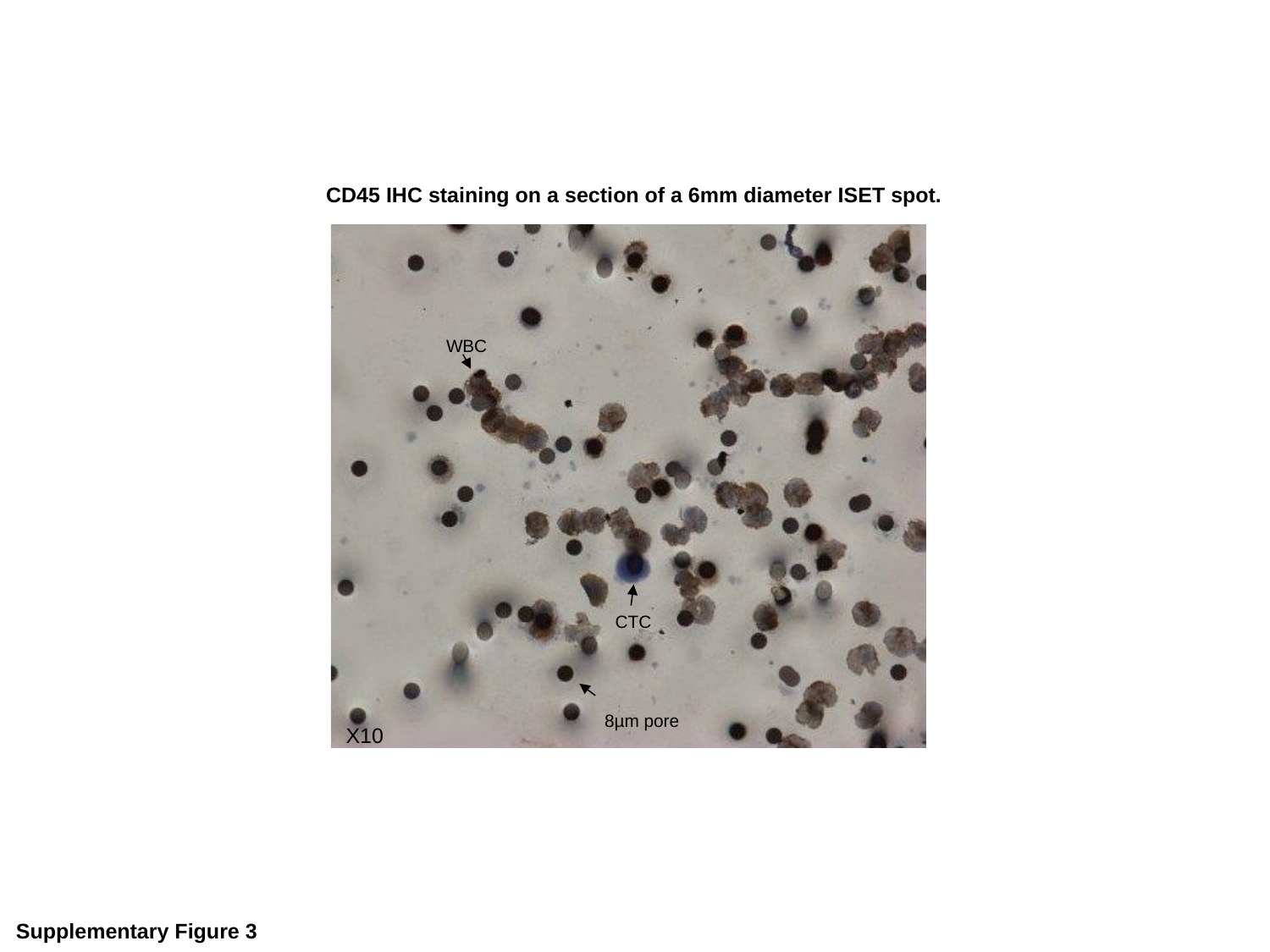

CD45 IHC staining on a section of a 6mm diameter ISET spot.
WBC
CTC
8µm pore
X10
Supplementary Figure 3

## Slide 4
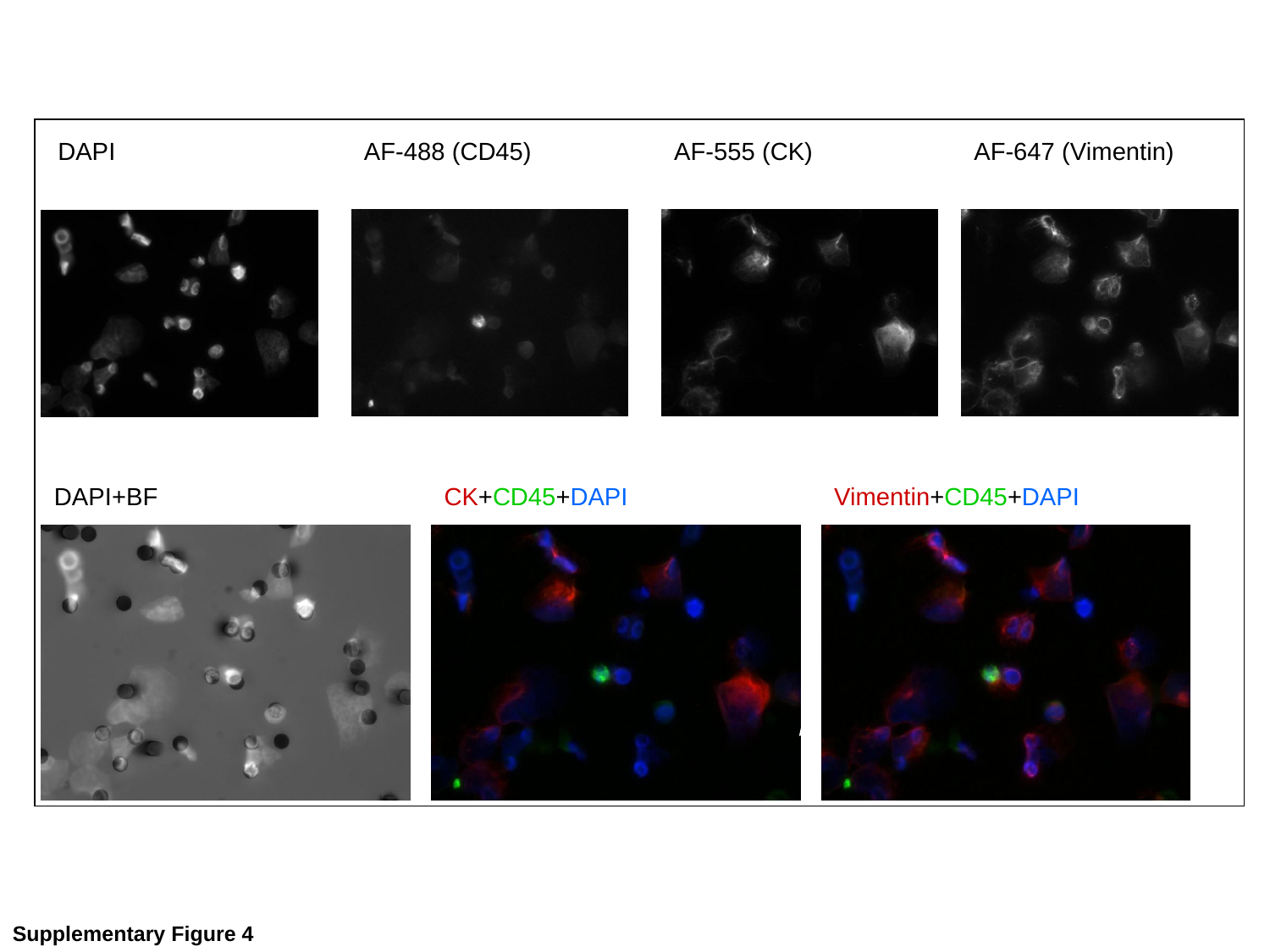

DAPI
AF-488 (CD45)
AF-555 (CK)
AF-647 (Vimentin)
DAPI+BF
CK+CD45+DAPI
Vimentin+CD45+DAPI
Supplementary Figure 4
