# Supplementary material for: A pilot study to explore circulating tumour cells in pancreatic cancer as a novel biomarker
Source: Br J Cancer. 2011 Dec 20;106(3):508–16. doi: 10.1038/bjc.2011.545 (PMC3273340; doi:10.1038/bjc.2011.545)
Supplement: Supplementary materials [file bjc2011545x2.doc]

**Supplementary Material**

**Assay development for assessment of CK and Vimentin in pancreatic cancer CTCs**

Panc-1 cells were cultured in Dulbecco’s Modified Eagle’s medium (DMEM containing 4.5g/500ml glucose, L-glutamine and pyruvate, (Invitrogen)) supplemented with 10% heat inactivated FBS. All cells were cultured at 37^0^C in a humidified incubator containing 5% CO2 and harvested at 80% confluence. The cell line was authenticated (STR DNA profiling) and was mycoplasma negative. Cells were harvested by trypsinization and 100µl single cell suspension in PBS containing ~ 10^3^ was spiked into 10ml of healthy volunteer blood for ISET filtration. ISET membranes were hydrated for 5 minutes in PBST (phosphate buffered saline with 0.1% tween-20) before placing in 0.2% Triton for 2 minutes. Membranes were then washed and incubated in 3% bovine serum albumin for 15 minutes. The following primary antibodies were then applied to the washed membranes; anti-pancytokeratin alexor-fluor 555 conjugated (1:50, mouse antibody clone c-11 #34/8s cell signaling), anti-Vimentin alexor-fluor 647(1:200 mouse antibody clone V9, #k1610 Santa Cruz) conjugated and anti-CD45 FITC conjugated (1:100, mouse antibody clone T29/33 Dako), for 45 minutes. Membranes were washed and mounted using prolong Gold with DAPI (Invitrogen p36935). Captured cells were visualized using an Olympus BX51 microscope (Tokyo, Japan) and an Uplan FLN x40 0.75 NO objective lens (the filter set used was the 89000 Sedat Quad - ET from Chroma (based in Vermont, USA). Image capture was achieved using a Coolsnap HQ camera (Photometrics, Tuscon, AZ, USA) and Metamorph software (Molecular Devices, Sunnyvale, CA, USA).

**Supplementary Figures**

**Figure 1**

**Controls for Immunohistochemical staining of markers used to characterise pancreatic cancer CTCs.**

**Top panel**. Cytospin of human pancreatic cell line HUPT4 stained for; **A**. cytokeratin 7, **B**. E-Cadherin, **D**. pan cytokeratin (c-11), **E**. EpCam, **F**. CD45 **C**.Cytospin of human pancreatic cancer cell line Panc-1 Vimentin. Middle panel. Cytospin of leucocytes for each of the above markers.

**Bottom panel**. **G**. Colon cancer SW620 cell pellet used as a positive control and melanoma cell line WM6224 cell pellet used as a negative control for EpCam tumour block IHC. **F**. Colon cancer SW620 cell pellet used as a positive control and melanoma cell line WM6224 cell pellet used as a negative control for pan cytokeratin (c-11) tumour block IHC.

**Figure 2**

**Cell spiking experiments to determine the optimum number of ISET spots for analysis in CTC enumeration by ISET.**

Tumour cells were spiked into 10ml of blood collected in an EDTA tube: 200 cells were spiked into 10mls healthy volunteer blood (Panel A); 400 cells were spiked into 10mls blood (Panel B). The blood sample was processed through the ISET platform and the resulting 10 spots were stained by IHC for CD45 to negatively select out tumour cells. Enumeration was determined for each of the 10 spots and the number of CTCs recovered per spot is given in the left hand column below the experiment number. All possible combinations of CTC number/spot was determined in excel and the mean determined for each of these combinations. Values shown are Mean ±SE for each of the combinations of CTC counts per ISET spot; SE reached a plateau once 4 spots or more were combined. Thus 4 ISET spots were analysed to give an average count per ml of blood which was extrapolated to 7.5ml blood allowing direct comparison with CellSearch.

**Figure 3**

**Four colour immunofluorescence of Panc-1 cells spiked into human healthy volunteer blood and filtered by ISET**

To demonstrate our future approach to a more detailed evaluation of CTCs, a four colour assay has been developed which combines the analysis CK and vimentin, with nuclear staining by DAPI and negative selection of CD45 positive leukocytes. Top row: single fluorophore images are shown on the top row. Bottom row, far left, shows the bright field image with DAPI staining where the dark circles are filter pores and the brightest DAPI stained cells are located directly over the pores. Bottom row middle image, composite pseudo colour image, CK red, CD45 green and DAPI blue and right image, composite pseudo colour image, Vimentin red, CD45 green and DAPI blue, identifying small white blood cells (green) and larger panc-1 tumour cells (which differentially express vimentin and CK).

**Figure 4**

**Patient ISET spot stained with CD45 for enumeration (negative selection) of CTCs.**

CD45 positive cells (leukocytes) were stained brown and circulating tumour cells were counter-stained with haematoxylin. This image represents approximately 0.45 µm of the 6mm individual ISET spot stained for this patient. The main contaminants are leukocytes.
